# Simultaneous non-union of scaphoid and capitate: a case report

**DOI:** 10.1080/23320885.2019.1613157

**Published:** 2019-06-22

**Authors:** Ryunosuke Fukushi, Kohei Kanaya, Kousuke Iba, Toshihiko Yamashita

**Affiliations:** Department of Orthopaedic Surgery, Sapporo Medical University School of Medicine, Sapporo, Japan

**Keywords:** Capitate, non-union, scaphoid

## Abstract

A 44-year-old painter separately developed simultaneous nonunions at the middle of the capitate and distal third of the scaphoid, for which we performed a cancellous bone graft from the iliac crest and a pedicled 1,2-intercompartmental supraretinacular artery graft, respectively. Union of both bones was ultimately achieved.

## Introduction

A combination of fracture scaphoid and capitate fractures is known as the scaphocapitate syndrome, which is a variety of trans-scaphoid, trans-capitate perilunar fracture-dislocation. [1] Scaphocapitate syndrome results from severe trauma caused by fall from a height or vehicular accidents and is characterised by a 90 to 180 degrees rotation of the head of the capitate. [2,3] However, there have been no reports of simultaneous nonunions of the scaphoid and capitate.

We report a case of a 44-year-old painter with simultaneous non-union of the scaphoid and capitate and describe the mechanism underlying the injury, treatment, and outcome.

## Case report

**Figure 1.**
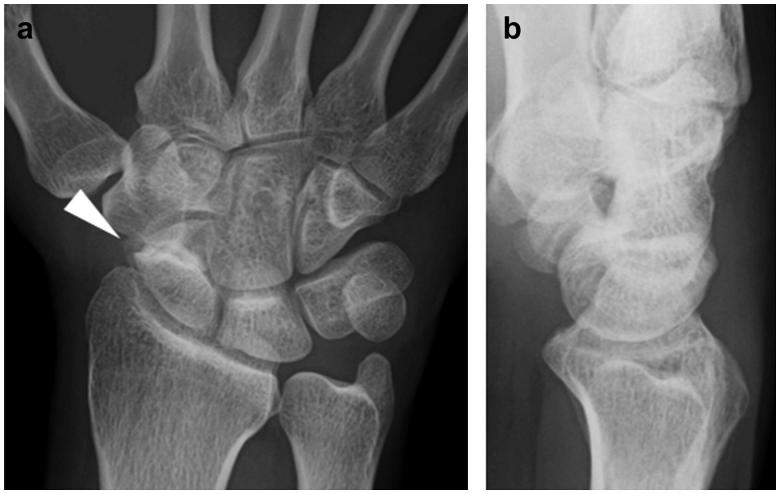
First radiographic assessment of the hand. (a) Posteroanterior and (b) lateral plain radiographs showing only scaphoid non-union.

A 44-year-old painter fell from a height of 5 metres 3 years prior to his referral to our clinic. Immediately after this fall, he visited a local clinic, where non-union of the scaphoid was noticed, but the capitate fracture was overlooked on radiographs (Figure 1). The patient was only treated with a plaster slab. Seven months after the trauma, the patient visited the clinic again due to persistent wrist pain. The radiographs showed non-union of not only the scaphoid but also the capitate. He was then referred to our clinic.

**Figure 2.**
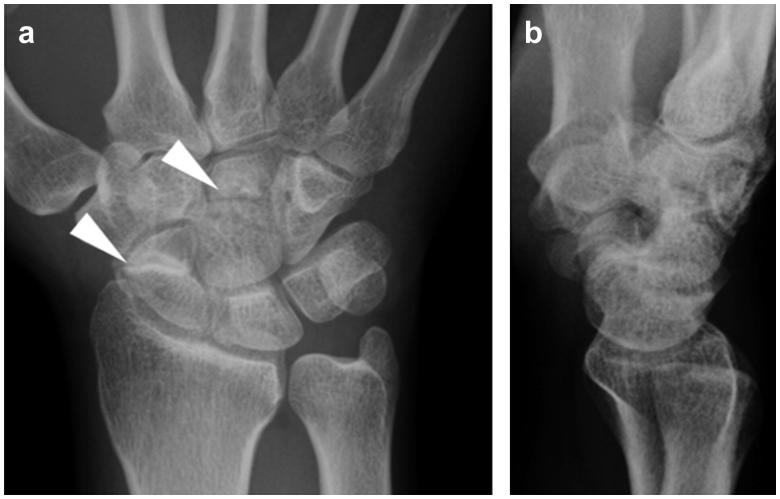
Radiographic assessment of the hand at our hospital. (a) Posteroanterior and (b) lateral plain radiographs showing scaphoid non-union and a radiolucent line in the central area of the capitate.

**Figure 3.**
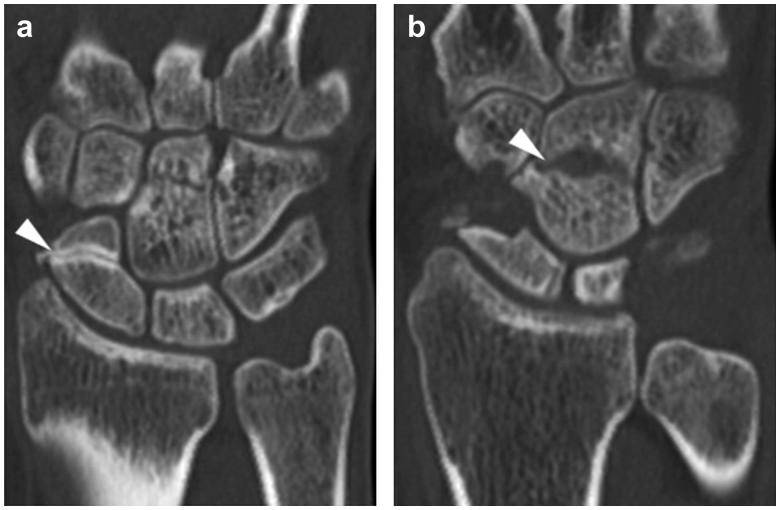
Computed tomography (CT) scan of the hand. Sagittal CT image of bone separation with osteosclerosis in the distal part of the scaphoid bone (a), and a radiolucent line in the central area of the capitate bone (b).

**Figure 4.**
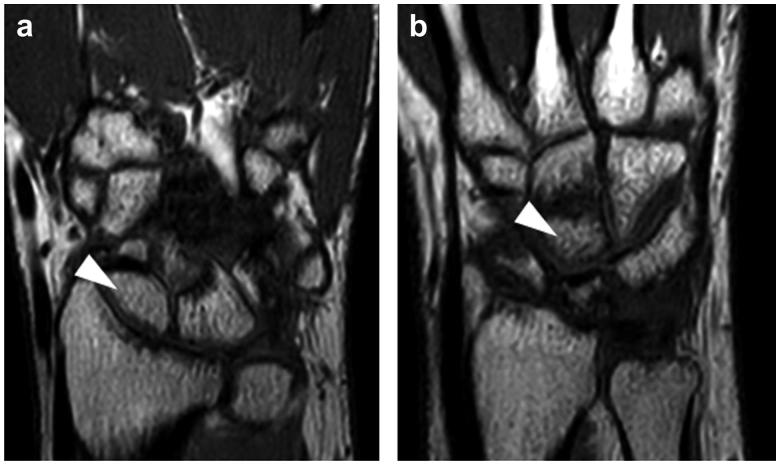
Magnetic resonance imaging (MRI) of the hand. T1-weighted MRI images showing proximal bone chips in both the scaphoid (a) and capitate (b) in high brightness. No impeded blood flow or necrosis was observed.

Examination revealed tenderness over his capitate bone; his visual analogue scale (VAS) score was 82 out of a total of 100 points, and Mayo wrist score 50 points. The range of motion of the wrist joint had 75° dorsiflexion and 50° palmar flexion. Radiographs and computed tomography (CT) showed non-union at the distal third of the scaphoid and at the middle of the capitate (Figures 2 and 3). T1-weighted magnetic resonance images (MRI) showed no avascular necrosis of the proximal pole of the scaphoid and capitate (Figure 4). We planned a cancellous bone graft from the iliac crest for the capitate non-union and a pedicled 1,2-intercompartmental supraretinacular artery graft (1,2 ICSRA) for the scaphoid non-union.

A transverse incision was made on the dorsal wrist. The capitate was exposed through capsulotomy of the midcarpal joint. After curettage of the capitate non-union, the cancellous bone graft from the iliac crest was performed, and the capitate was fixed with an Acutrak Mini (Acutrak Headless Compression Screw System, Japan Medicalnext Co., Ltd., Tokyo, Japan). A gentle curvilinear dorsoradial incision was used to expose the scaphoid and bone graft donor site. The dorsoradial capsule of the wrist joint was incised, and the non-union was removed from the sclerotic fracture surfaces and from the exposed cancellous bone. The 1,2 ICSRA was visualised on the surface of the retinaculum between the first and second extensor tendon compartments. Both compartments were opened, and a cuboid bone graft was raised on the 1,2 ICSRA. After releasing the tourniquet, circulation of the bone graft was confirmed. The pedicle bone graft was trimmed to fit the defect and was placed in the non-union bed using two K-wires. Postoperatively, the wrist was immobilised with a below-elbow plaster cast for 12 weeks.

**Figure 5.**
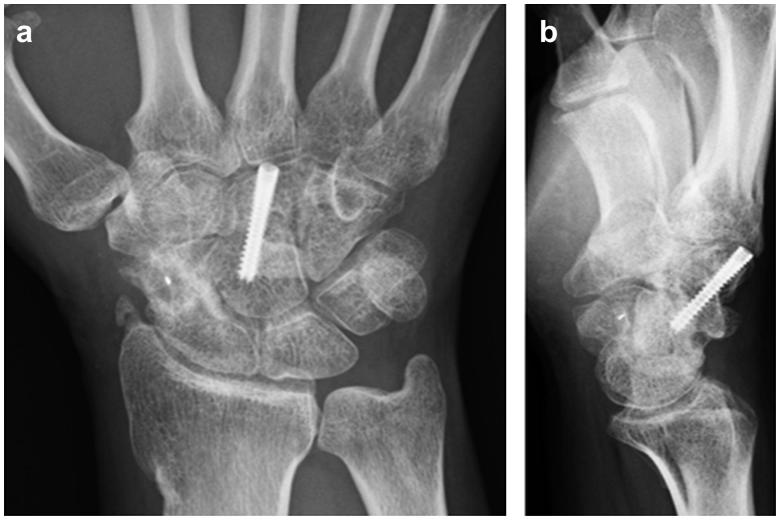
Postoperative radiographic assessment of the hand. Radiographs showing the healing of both nonunions without evidence of arthritic changes.

Union of both the capitate and scaphoid bones was achieved at 2 and 3 months after surgery, respectively, and the K-wires in the scaphoid bone were removed at 3 months post-surgery. One year after surgery, the wrist was painless, and the range of motion of the wrist joint had 55° dorsiflexion and 60° palmar flexion, along with a Mayo wrist score of 70 points. Radiography performed 1 year after surgery showed healing of both nonunions, without evidence of arthritic changes (Figure 5).

## Discussion

To our knowledge, this is the first report of scaphoid and capitate non-union existing simultaneously. From the history of the patient’s visits to local clinics, these two nonunions developed separately due to the scaphoid non-union being neglected and the capitate fracture being overlooked. In other words, the capitate fracture had occurred in the presence of the scaphoid non-union. Further, the patient sustained a high-energy injury after falling from a height of 5 metres.

Capitate fracture occurs in the following three situations: isolated fracture, perilunate dislocation, and scaphocapitate syndrome. Our patient experienced non-union following capitate fracture as a complication of scaphoid non-union. Although the pathology was associated with the scaphoid and capitate bones and was similar to that of scaphocapitate syndrome, the mechanism of occurrence was considered to differ from that of this syndrome.

The scaphocapitate syndrome is a combination of fractures of the scaphoid and capitate and occurs after a high-energy trauma of the wrist. [4] The syndrome results from wrist hyper-extension: the dorsal end of the radius can hit the capitate directly, and the continued movement leads to a rotation of 90 degrees from the proximal pole of the capitate and an additional rotation of 90 degrees during return to the neutral position. [2,3]

In the present case, the capitate fracture had occurred at the middle portion, suggesting differences in the mechanism of occurrence between scaphocapitate syndrome and the condition in our case. We considered the following mechanism underlying the middle third fracture of the capitate. The transverse axis of the radius rotation might have transferred from the lunate to the proximal fragment of the scaphoid due to the non-union of the distal third of the scaphoid. Then, the lever arm from the rotation axis of the wrist to the radius might have moved farther, and the dorsal end of the radius might have hit the middle of the capitate, thus, resulting in a capitate fracture. The proximal fragment of the capitate might not have rotated because the fragment was larger.

Apergis et al. [4] reported six patients with scaphocapitate syndrome. In one of these patients who had a combination of a scaphoid non-union and capitate fracture, the scaphoid non-union did not unite despite treatment with a cancellous bone graft and Herbert screw. In our patient, a vascularised bone graft was performed for the scaphoid non-union due to risk of failure for the cancellous bone graft, as reported by Apergis et al. [4] As a result, the scaphoid non-union was resolved 3 months after surgery.

Jethanandani et al. [5] conducted a review of surgical treatment and conservative treatment using casts and braces for capitate fractures and reported that with surgical treatment being superior, immobilisation using rigid internal fixation and autologous bone fragments are effective. In addition, retrograde screw fixation is considered ideal according to the hemodynamics of the capitate bone. In our patient, we achieved bone union of the capitate through internal fixation similarly using retrograde screws and iliac bone transplantation.
